# Nesting behaviour influences species-specific gas exchange across avian eggshells

**DOI:** 10.1242/jeb.103291

**Published:** 2014-09-15

**Authors:** Steven J. Portugal, Golo Maurer, Gavin H. Thomas, Mark E. Hauber, Tomáš Grim, Phillip Cassey

**Affiliations:** 1 Structure and Motion Laboratory, The Royal Veterinary College, University of London, North Mymms, Hatfield, Herts AL9 7TA, UK; 2 School of Earth and Environmental Sciences, University of Adelaide, SA 5005 Australia; 3 Department of Animal and Plant Sciences, University of Sheffield, Sheffield S10 2TN, UK; 4 Department of Psychology, Hunter College and the Graduate Center of the City University of New York, 695 Park Avenue, New York, NY 10065, USA; 5 Department of Zoology and Laboratory of Ornithology, Palacký University, Olomouc, CZ-771 46 Czech Republic

**Keywords:** Avian eggshells, Life history, Museum specimens, Nest environment, Permeability

## Abstract

Carefully controlled gas exchange across the eggshell is essential for the development of the avian embryo. Water vapour conductance (*G*_H_2_O_) across the shell, typically measured as mass loss during incubation, has been demonstrated to optimally ensure the healthy development of the embryo while avoiding desiccation. Accordingly, eggs exposed to sub-optimal gas exchange have reduced hatching success. We tested the association between eggshell *G*_H_2_O_ and putative life-history correlates of adult birds, ecological nest parameters and physical characteristics of the egg itself to investigate how variation in *G*_H_2_O_ has evolved to maintain optimal water loss across a diverse set of nest environments. We measured gas exchange through eggshell fragments in 151 British breeding bird species and fitted phylogenetically controlled, general linear models to test the relationship between *G*_H_2_O_ and potential predictor parameters of each species. Of our 17 life-history traits, only two were retained in the final model: wet-incubating parent and nest type. Eggs of species where the parent habitually returned to the nest with wet plumage had significantly higher *G*_H_2_O_ than those of parents that returned to the nest with dry plumage. Eggs of species nesting in ground burrows, cliffs and arboreal cups had significantly higher *G*_H_2_O_ than those of species nesting on the ground in open nests or cups, in tree cavities and in shallow arboreal nests. Phylogenetic signal (measured as Pagel's λ) was intermediate in magnitude, suggesting that differences observed in the *G*_H_2_O_ are dependent upon a combination of shared ancestry and species-specific life history and ecological traits. Although these data are correlational by nature, they are consistent with the hypothesis that parents constrained to return to the nest with wet plumage will increase the humidity of the nest environment, and the eggs of these species have evolved a higher *G*_H_2_O_ to overcome this constraint and still achieve optimal water loss during incubation. We also suggest that eggs laid in cup nests and burrows may require a higher *G*_H_2_O_ to overcome the increased humidity as a result from the confined nest microclimate lacking air movements through the nest. Taken together, these comparative data imply that species-specific levels of gas exchange across avian eggshells are variable and evolve in response to ecological and physical variation resulting from parental and nesting behaviours.

## INTRODUCTION

The striking diversity in shape, size and pigmentation of avian eggs (Hauber, 2014) provides an ideal model system for studying the causes and consequences of evolutionary diversity and adaptive function. The avian eggshell is a complex, multifunctional bioceramic (Fernandez et al., 1997). It actively shapes the developmental milieu of the embryo by protecting it from mechanical damage, facilitating gas exchange and providing calcium for bone growth (Ar et al., 1974; Maurer et al., 2011). Gas exchange across the shell relies on the diffusive properties of the eggshell and the environmental conditions in which the egg is placed, and is vital for the development of the embryo within the egg (Ar and Rahn, 1978; Ar and Rahn, 1980; Vleck et al., 1983; Rahn and Paganelli, 1990). Gas exchange contributes to the rate of water loss, estimated across the eggshell as water vapour conductance (*G*_H_2_O_; mg day^−1^ Torr^−1^), which must be mediated in such a way that desiccation does not endanger the embryo, while sufficient water is lost for embryo growth and air cell formation (Ar and Rahn, 1980; Barrott, 1937; Romijn and Roos, 1938).

As birds breed in almost all terrestrial environments, including habitats with extreme levels of humidity, altitude and temperature (Lomholt, 1976; Sotherland et al., 1980; Davis et al., 1984; Davis and Ackerman, 1985; Arad et al., 1988; Carey et al., 1989; Carey et al., 1990; Walsberg and Schmidt, 1992; Carey, 1994), the structure of the eggshell is likely to play an important role in allowing bird species to successfully expand into and inhabit a wide variety of habitats. To fully understand the diversity of avian eggshell structure requires an analysis of the evolutionary basis of the structural adaptations for eggshells' gas exchange in different environments and nesting conditions, and across species with varying life histories (e.g. Portugal et al., 2014). Because all nutrients for embryonic development are deposited by the avian mother into the egg prior to laying, suitable levels of gas exchange and parental modulation of incubation temperatures constitute the only physical control of the requirements for embryonic development in birds (Ar et al., 1974; Hoyt et al., 1979; Paganelli, 1980; Visschedijk, 1980; Booth and Seymour, 1987). Here we examine how broad-scale evolutionary and ecological variation, species-specific breeding behaviour and phylogenetic relatedness can explain variation in gas transfer across the avian eggshell.

Quantifying patterns of interspecific variability in *G*_H_2_O_ and the associated egg-mass loss across phylogenetically diverse taxa is essential to understand how flexibly birds have adapted to their diverse breeding environments. Typically, studies of eggshell *G*_H_2_O_ have focused on closely related species and family groups of birds,

**Table 1.**
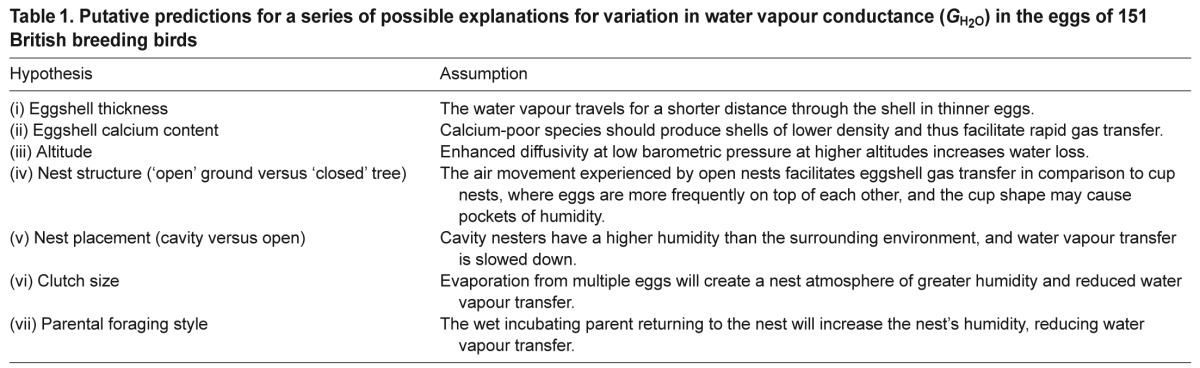
Putative predictions for a series of possible explanations for variation in water vapour conductance (*G*_H_2_O_) in the eggs of 151 British breeding birds

in an attempt to elucidate the ultimate and proximate causes of variation in the *G*_H_2_O_ between related species (e.g. Vleck et al., 1983). Although the potential effects of nest environment and nest structure on *G*_H_2_O_ have both been studied intensively, a focus on closely related taxa, even in comparative studies, means that potential confounds of shared phylogenetic affinities and life-history traits, which may play parallel or contrasting roles in determining the optimal *G*_H_2_O_, have not yet been identified (Cassey et al., 2010). Therefore, we measured surface-specific *G*_H_2_O_ in the eggs of a broad taxonomic spectrum of 151 British breeding bird species spanning several orders, using a repeatable and standardised methodology. We tested ecological hypotheses on how modifications in the *G*_H_2_O_ in the eggs of different lineages vary with respect to differences in the humidity and pressure of different nest environments. Based on extensive previous literature, we tested several physical and life-history variables that may explain variation in *G*_H_2_O_ across species (detailed in Table 1). These can broadly be grouped into three categories: egg structure (predictions i and ii in Table 1), nest habitat and type (predictions iii–v) and life-history traits of the adult birds (predictions vi and vii).

## RESULTS

### Reliability of *G*_H_2_O_ measurements

Mass loss between subsequent weighing sessions was highly repeatable for each eggshell fragment (Pearson's *r*=0.99, *n*=1281) and contributed to less than 5% of the total variability in *G*_H_2_O_ between eggs. Nested ANOVA indicated that <50% of the variability in *G*_H_2_O_ among individual eggs was explained through differences between the three eggshell regions: the blunt end (B), equator (E) and pointed end (P). However, the analysis of these three eggshell regions independently showed that individual egg (Egg ID) contributed 53.8, 59.6 and 67.0% of the total variability in *G*_H_2_O_ for B, E and P, respectively.

**Fig. 1.**
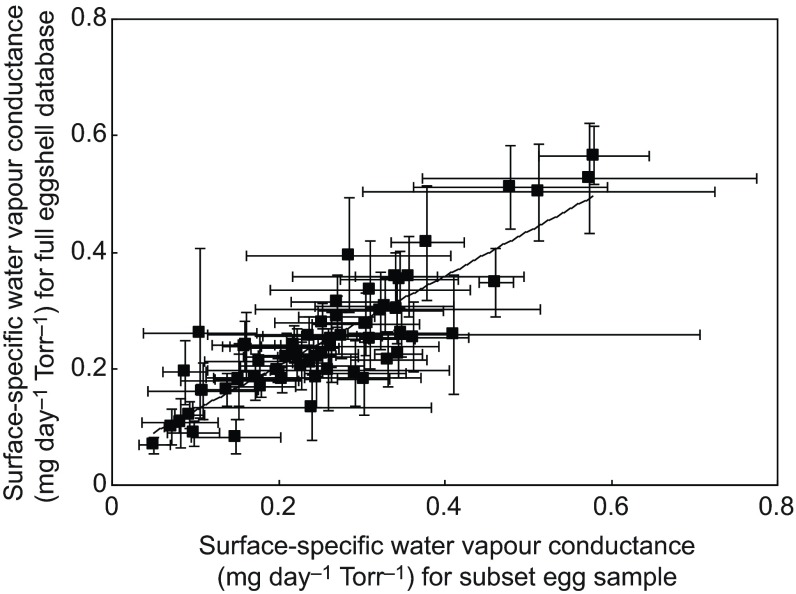
**Mean (±s.e.m.) surface-specific water vapour conductance (*G*_H_2_O_) for 62 British breeding bird species, sourced from a single museum donor, compared with the *G*_H_2_O_ for the same species measured in eggs from multiple different donors.** Analysis showed the values of *G*_H_2_O_ to be highly repeatable for a species, and that egg donor origin was not a significant factor in the determination of average *G*_H_2_O_ for a species. Values of *G*_H_2_O_ for the three segments are combined (blunt end, equator and pointed end).

To investigate further the contribution of individual variation in eggshell collection and preparation to the variation in *G*_H_2_O_, we analysed a subset of eggs that were donated to the Natural History Museum, Tring, as a single source collection compiled by a single collector. This subsequent analysis of the single largest collection of eggs from one donor (62 species and 42% of the collection in total) demonstrated that when analysed collectively, region (B, E and P), i.e. within-egg variability, was responsible for the largest percentage of variability (39.7%) in *G*_H_2_O_ between eggs. In contrast, when the regions were separated, Egg ID was only responsible for 16.5, 9.6 and 2.4% of the variability between eggshells for B, E and P, respectively, with 77% of the variability in *G*_H_2_O_ being explained by phylogenetic effects owing to species differences, within the same avian families. From these results, we inferred that our methods are indeed sufficient to detect significant differences between species, and that an average species value’ across all collections of *G*_H_2_O_ is both obtainable and highly repeatable (Fig. 1).

### Differences in *G*_H_2_O_ across eggshells

There was a significant and consistent difference in the *G*_H_2_O_ between the three eggshell regions (*F*2,1281=20.9, *P*<0.001). For the majority of species (>80%), the B region, coinciding with the eventual location of the air cell, had a significantly lower *G*_H_2_O_ compared with both E and P regions (*F*2,1281=25.76, *P*<0.001). Mean values of *G*_H_2_O_ for E and P were highly correlated (*r*=0.65, *n*=144, *P*<0.001), and not significantly different from each other (0.2559±0.08 and 0.2532±0.09 mg day^−1^ Torr^−1^ for E and P, respectively, B=0.2245±0.07 mg day^−1^ Torr^−1^). Consequently, we considered the B and E regions only in subsequent analyses (all data are available in supplementary Tables S1 and S2).

### Phylogenetic correlation

For all eggshell regions (i.e. whole eggshell or B and E regions separately), Pagel's λ values were intermediate between 0 and 1 and

**Table 2.**
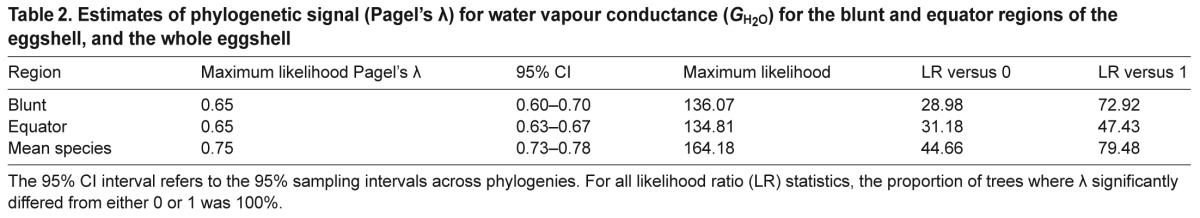
Estimates of phylogenetic signal (Pagel's λ) for water vapour conductance (*G*_H_2_O_) for the blunt and equator regions of the eggshell, and the whole eggshell

significantly different from both (Table 2), suggesting that some phylogenetic signal is retained in *G*_H_2_O_. A Pagel's λ value of 0 indicates that values of the *G*_H_2_O_ trait are independent of phylogeny, while a Pagel's λ value of 1 indicates that values of *G*_H_2_O_ are evolving according to Brownian motion on the given phylogeny (e.g. Cassey et al., 2010). Intermediate values of Pagel's λ imply that traits have evolved according to a process in which the effect of phylogeny is weaker than in the Brownian model. These patterns were detected regardless of whether life-history traits were included in the phylogenetic model (Table 2).

**Fig. 2.**
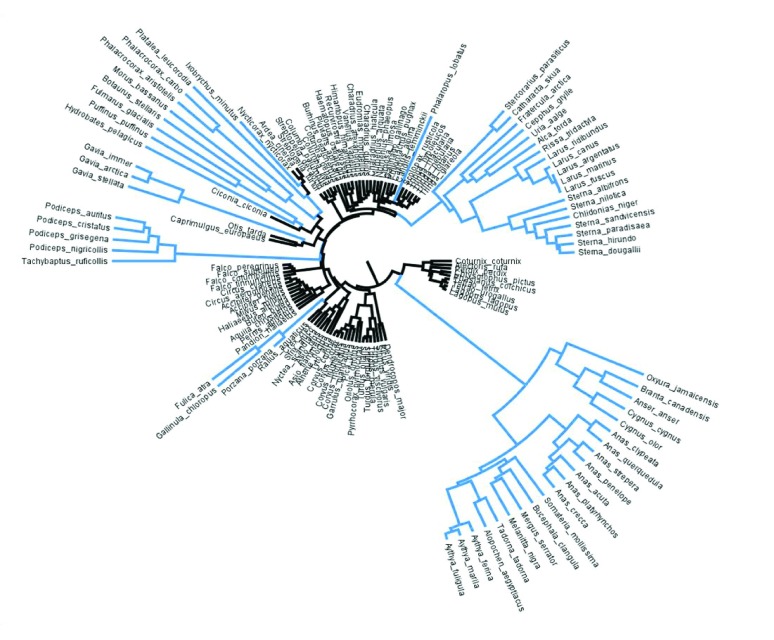
**Phylogenetic tree of the 151 species of British breeding birds.** Those species which are classified ‘wet parents’ (habitually return to the nest wet) are coloured blue. The remaining species (coloured black) are ‘dry parents’. The branch length are proportional to the rate of diversification in *G*_H_2_O_, which was ~5.5 times greater in the wet incubating parents group when compared with those species that return to the nest dry.

**Fig. 3.**
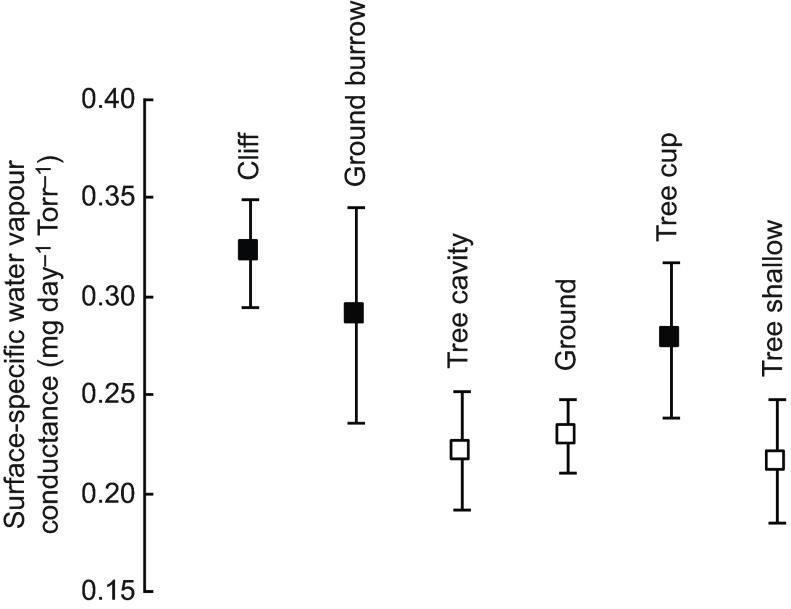
**Mean (±s.e.m.) water vapour conductance (*G*_H_2_O_) for six nest types and nest locations, measured in 151 species of British breeding birds.** Nest types/locations indicated with filled squares had significantly higher *G*_H_2_O_ values than those indicated with open squares.

Of our 17 life-history and habitat variables, only two were retained in the final model of *G*_H_2_O_ variation across species. Using mean *G*_H_2_O_ values for the whole eggshell of each species, the model that best explained the variation in *G*_H_2_O_ (*r*^2^=0.17, *F*5,132=5.38, *P*<0.001) retained only wet incubating parent (*F*1,132=6.08, *P*<0.001; Fig. 2) and nest type (*F*5,132=4.99, *P*<0.001; Fig. 3) as significant predictor variables. The relative contribution of the non-significant variables included in the initial full model, which were removed, are presented in supplementary material Table S3.

Species with wet incubating parents had a *G*_H_2_O_ that was, on average, 0.042 mg day^−1^ Torr^−1^ higher than those species where parents do not return to the nest wet. The rate of diversification in *G*_H_2_O_ was approximately five times greater between species in which parents incubate with wet plumage than it was between species in which parents incubate with dry plumage [as represented by the branch lengths in Fig. 2, relative rate estimate=4.72 (2.94–67.95% CI), likelihood ratio test=39.93, d.f.=1, *P*<0.001]. Eggs of species nesting in ground burrows, cliffs and arboreal cups had significantly higher *G*_H_2_O_ than those nesting on the ground in open nests or cups, in tree cavities and in shallow arboreal nests (Fig. 3).

For *G*_H_2_O_ of the B region only, nest type (*F*5,132=4.48, *P*<0.001) and wet parent (*F*1,132=11.86, *P*<0.001) were the only significant predictor variables retained in the model (*r*^2^=0.18, *F*5,132=5.96, *P*<0.001). For the E region only, nest type (*F*5,132=4.93, *P*<0.001) and average eggshell thickness (*F*1,132=7.22, *P*<0.001) were the only significant predictor variables retained (*r*^2^=0.17, *F*5,132=5.38, *P*<0.001), with an increase in thickness (estimate ± s.e.m.=0.16±0.05) resulting in an increase in *G*_H_2_O_.

## DISCUSSION

Differences observed in the rate of water vapour conductance (*G*_H_2_O_) across the avian eggshell covary with a combination of both shared ancestry (phylogenetic relatedness) and several life-history and ecological traits (Table 2). Species-specific behavioural and environmental parameters can play an important role in influencing *G*_H_2_O_ (Deeming, 2002), and our study discovered that nest type and whether the incubating parent returns to the nest wet were the only statistically significant factors that predicted interspecific variability

in *G*_H_2_O_ of bird eggs in a large sample of representative British species.

Whereas *G*_H_2_O_ is vital for successful incubation, nest site choice for the adult bird will likely involve a trade-off between a suitable nest microclimate for optimum egg-water loss and minimising nest predation risks, the latter being the most important predictor of nesting success across birds (Ricklefs, 1969). The full effects on embryonic development, hatching success, post-hatching growth and overall fitness of sub-optimal egg-water loss are not understood. However, it is clear that eggs and clutches incubated under conditions that lead to sub-optimal water loss have reduced hatching success (e.g. Buhr, 1995; Yildirim and Yetisir, 2004). Yet, the long-term effects of water loss on fledgling success, when the eggs hatch, remain unknown, especially because water loss is likely to covary with fluctuations in incubation temperature, which in turn has crucial fitness impacts on post-hatching development (Pérez et al., 2008; DuRant et al., 2010).

Importantly, in our results clutch size and developmental mode were not significant main predictor variables of gas transfer across our relatively limited geographic sampling of British bird eggs (see Carey, 1994; Deeming, 2002). We originally hypothesised (see Table 1) that species with large clutch sizes would have higher *G*_H_2_O_ than those species with relatively smaller clutches, in response to the increased humidity that a greater number of eggs present in the nest might produce. However, behaviours of the incubating adult bird, such as egg turning using the feet (Morgan et al., 2003; Skutch, 1976), beak (Haftorn, 1994; Handrich, 1989) and tremble-thrusting (Vleck, 1981), may effectively mix the air and reduce the chance of pockets of humidity developing over time, so that the eggs of species with large clutches do not require an adaptation to their *G*_H_2_O_.

Our study helps clarify to what extent the incubating parent influences the *G*_H_2_O_ in the developing egg. We have shown that both the behaviour of the incubating adult (returning to the nest with wet or dry plumage) and its choice of nest site can affect the rate of gas exchange across the eggshell. Previously it was found that the adult bird can contribute to the control of the nest microclimate, and as much as 30–45% of moisture in the nest's atmosphere is provided by the incubating parent (e.g. Andersen and Steen, 1986). This percentage would be even greater if the eggs and nest are in a confined area such as a burrow, cavity or cupped nest. Our results are consistent with previous studies that revealed significantly higher *G*_H_2_O_ in species that use underground burrow nests and cup nests (Fig. 3) (Rahn et al., 1977; Ackerman and Platter-Reiger, 1979; Carey, 1980; Rahn and Hammel, 1982). However, the result reported here, namely that the *G*_H_2_O_ of eggs found in ground-nesting species and cavities was lower than these aforementioned groups, is unexpected (see Deeming, 2002). In the considerably smaller phylogenetic sample of bird species (seven members of the order Pelecaniformes, and three members of the order Charadriiformes) used by Vleck et al. (Vleck et al., 1983), ground-nesting birds typically laid eggs with a high rate of *G*_H_2_O_, potentially to overcome the lack of wind and air movements, which can result in a humid nest microenvironment. Similarly, eggs of cavity-nesting birds studied previously show comparable traits of increased *G*_H_2_O_.

Measuring the *G*_H_2_O_ across the eggshell under standard conditions reveals adaptations that facilitate optimal water loss during incubation in very different nest environments. To achieve optimal water loss, the *G*_H_2_O_ is likely to be very similar between all species under typical nest conditions. Only under standard laboratory conditions will these differences in *G*_H_2_O_ due to structural adaptations of the avian eggshell become apparent. Measuring the *G*_H_2_O_ under standard conditions, however, may result in certain functional differences between the eggs of particular species being missed. For example, the eggs of eared grebes (*Podiceps nigricollis*) are covered in rotting vegetation when the incubating parents make recesses, and frequently can be partially submerged in water (Davis et al., 1984; Lomholt, 1984; Board, 1982; Sotherland et al., 1984). The extreme changes in humidity and temperature associated with this nest environment may mean that the *G*_H_2_O_ of grebe eggs is more flexible and responsive to changes in the environment, and the eggs more resilient to desiccation and wetting, thus being able to cope with wider temperatures and humidity ranges. Therefore, it may be that under standard conditions, eggs of species that are better adapted and equipped to cope with changes in environmental factors (e.g. temperature and humidity) will not exhibit these differences in physical properties.

It would be worthwhile for future studies to test the *G*_H_2_O_ in eggs of certain species under different temperatures and humidity ranges, or specifically replicating particular nesting microhabitats, to ascertain a thermal and external humidity tolerance zone of *G*_H_2_O_ as they are related to species-specific environmental factors and behavioural patterns during incubation.

## MATERIALS AND METHODS

### Egg samples and preparation

Eggs of 151 British breeding bird species were obtained from the destructive collection of the Natural History Museum, Tring (UK) (Cassey et al., 2010; Cassey et al., 2012; Maurer et al., 2014; Portugal et al., 2010a; Portugal et al., 2010b; Russell et al., 2010). Previously, we demonstrated that *G*_H_2_O_ of museum and fresh eggs of the same species does not differ significantly (Portugal et al., 2010a). Eggshell parameters [length (mm), breadth (mm), mass (g) and thickness (μm)] were measured directly on the sampled eggs. Only species with an eggshell >30 mm in length and >0.075 mm thick were included, as smaller shells were unsuitable for reliable *G*_H_2_O_ measurements (see Portugal et al., 2010a).

Three different regions of the eggshell were used for *G*_H_2_O_ measurements: the blunt end (B), equator (E) and pointed end (P). These fragments of the eggshell (~225 mm^2^) were cut from the shell using a diamond-tipped dentist drill (Milnes Bros., Surrey, UK). Prior to *G*_H_2_O_ being measured, eggshell thickness was recorded using a Mitutoyo Series 227–203 constant measurement force micrometer (as described in Maurer et al., 2010; Maurer et al., 2012).

Detailed information about eggshell preparation and measurements of the *G*_H_2_O_ can be found elsewhere (Portugal et al., 2010a; see also Booth and Seymour, 1987; Maurer et al., 2011). Briefly, the eggshell fragments were glued to the top of Eppendorf tubes (surface area of 24.4 mm^2^) that had been previously filled with 200 μl of distilled water. The Eppendorf units were placed into desiccators (Camlab, Over, Cambridgeshire, UK), which in turn were housed in a constant-temperature thermocabinet (Camlab) at 30±1°C. Temperature, humidity and pressure were monitored continuously via a logtag analyser and an average was logged every 1 min (Loggershop, Bournemouth, Dorset, UK). After 24 h, the Eppendorfs were weighed (g) to four decimal places (Sartorius, Göttingen, Germany) before being returned to the desiccators. The Eppendorf tubes and eggshell fragments were weighed at the same time of day on three successive days to give two values of 24 h *G*_H_2_O_. Any mass loss was assumed to be the result of water loss (*sensu* Booth and Seymour, 1987). Calculation of *G*_H_2_O_ was conducted as previously described (Portugal et al., 2010a).

### Life-history and ecological data of a representative avian phylogenetic sample from Britain

Species were classified following Sibley and Monroe (Sibley and Monroe, 1990). Life-history and ecological data were gathered primarily from *Handbook of the Birds of the World* Volumes 1–13 (Del Hoyo et al., 1992-2010), and cross referenced with *Birds of the Western Palearctic* (Cramp et al., 1977-1994). In addition, supplementary data were obtained from family- and species-specific monographs, and field guides to nests (sources available on request).

The following variables were recorded from the literature: adult body mass (g), clutch size (modal number of eggs), incubation length (days), shared incubation between two parents (no/yes), median breeding range (degrees latitude), nest type [cup/non-cup (open)], nest concealed (no/yes), ground nesting (no/yes), arboreal nesting (no/yes), cavity nesting (no/yes), cliff nesting (no/yes), diet (calcium rich/herbivore), development mode (precocial/altricial), migration (no/yes), whether the parental foraging style meant that adults returned habitually to the nest with wet plumage (no/yes wet incubating parent variable; see Results), and general breeding habitat (open/closed) [see Cassey et al. (Cassey et al., 2010) for full description]. All variables in our data set could be assigned for all species.

Body mass of adult birds was taken as a mean for both sexes, primarily from the *Handbook of Avian Body Masses* (Dunning, 2007). Breeding latitude was compiled from data tabulated by Orme et al. (Orme et al., 2005; Orme et al., 2006). Nest type was recorded as evidence of cup building versus open shallow nests, based on the description in the literature. The open category includes shallow nests (e.g. oystercatcher, *Haematopus ostralegus*), and complete absence of nest [e.g. Eurasian stone curlew (*Burhinus oedicnemus*), common guillemot (*Uria aalge*)]. Further detail was then added on whether the nest was placed concealed in vegetation or camouflaged. This information was based on the location of the nest, the nest structure and its components (nest material). A nest in open view (0) was not considered hidden [e.g. osprey (*Pandion haliaetus*), pied avocet (*Recurvirostra avosetta*), common coot (*Fulica atra*)]; by contrast, a nest that is concealed in a crevice, in a burrow or by foliage was considered hidden (Ar and Rahn, 1978). The migratory nature of a species was determined from the illustrative maps in the *Handbook of the Birds of the World* (Del Hoyo et al., 1992-2010), by establishing whether the entire population underwent a full move from one region to another, and as such, did not include locally dispersing species and partial migrants, which were also scored as non-migratory. General breeding habitat was assigned based on the descriptions of McNaught and Owens (McNaught and Owens, 2002), where habitats were broadly defined as closed (closed woodland, reed beds, rank grass) or open (arid regions, grasslands, heathland, wooded grasslands, open woodland, marsh).

The average degree of maculation of the eggshell was determined by three observers from specimens studied at the Natural History Museum, Tring, collection [see Cassey et al. (Cassey et al., 2010) for repeatability estimates]. For each species, the eggs were assessed for presence and coverage of maculation using a three-point scoring system and points were averaged between observers to obtain a maculation score. Maculation was recorded as ‘0’ if the egg was immaculate, ‘1’ for maculation present but with a clear, dominant background colour, and ‘2’ for widespread maculation that covered the entirety of the egg (see also Brulez et al., 2014). The full species matrix of life-history traits and eggshell parameters can be found in supplementary material Tables S1 and S2.

### Phylogenetic methods and analysis

We revised and updated a recent phylogenetic hypothesis of British birds (Thomas, 2008). The phylogeny was based on sequence data from 12 protein-coding mitochondrial genes and included 151 British breeding bird species. The published tree was extended by: (1) adding sequence data for 15 more species, (2) increasing the number of genes included where available and, (3) replacing the data on Thomas' (Thomas, 2008) three surrogate species with recently published data on the focal species (little bittern, *Ixobrychus minutus*; European bee-eater, *Merops apiaster*; and European golden plover, *Pluvialis apricaria*). Each gene was aligned by eye in SE-AL v. 2.0a11 (Rambaut, 2002). All sequence data were collected from GenBank (Benson et al., 2007) using Geneious v. 4.8.5 (Drummond et al., 2009), and sequence accessions and full alignments are available on request.

We used BEAST 1.5.4 for phylogenetic analyses using a codon-specific GTR+Γ substitution model in which substitution rates, among-site rate variation and state frequencies at third codon positions were unlinked (GTR+CP_112_+Γ). We used a Yule prior on the branching process and an uncorrelated relaxed clock in which rate variation among branches was drawn from a log-normal distribution. We applied two topology constraints to the phylogeny by defining the monophyly of the widely accepted Neoaves and Galloanserae clades. Note that this is more liberal than the 11 constraints used by Thomas (Thomas, 2008) and allows us to better account for the uncertainty in topology in the deeper nodes of avian phylogeny. We conducted two runs, one each for 40 and 50 million generations, sampling trees every 10,000 generations. We assessed mixing within runs and convergence between runs using Tracer v. 1.5.0 (Drummond and Rambaut, 2007) based on visual inspection of traces and effective sample sizes of tree parameters (node ages of the two constrained nodes), posterior log-likelihoods and substitution model parameters. Both runs converged rapidly and we discarded 10% of generations from each run as burn-in. We combined the post-burnin samples of the two runs to yield a posterior distribution in which the majority of parameters had effective sample sizes of >500 (and all >100). For use in subsequent phylogenetic analyses (see below) we subsampled down to 1000 trees (drawn from the full posterior distribution of >8000 trees) and pruned each tree to the 49 species in the eggshell data set. We also extracted the maximum clade credibility tree from the full tree distribution for use as a single ‘best’ representative tree.

We estimated Pagel's λ (Pagel, 1997; Pagel, 1999; Shackleton et al., 2000), using the R-library motmot (available from http://r-forge.r-project.org/), as a measure of the strength of phylogenetic signal in the *G*_H_2_O_ variables. Pagel's λ varies from 0 to 1, where 0 indicates no phylogenetic signal in the data and 1 is consistent with a Brownian motion model of trait evolution in which the phylogeny accurately reflects the covariances between species for a given trait (for details, see Shackleton et al., 2000). To assess the effects of phylogenetic error, we repeated the λ fitting procedure with a distribution of 1000 phylogenetic hypotheses (see above).

We tested hypotheses on the correlates of *G*_H_2_O_ using the R-library CAIC (available from http://r-forge.r-project.org/) to fit phylogenetically controlled general linear models. Specifically, we used the function pglmEstLambda to fit Pagel's λ simultaneously with each regression model in order to appropriately correct for phylogenetic signal in the residuals. We first tested the correlation between *G*_H_2_O_ variables without any other covariates and repeated this over 1000 phylogenies. We then fitted full regression models including all relevant explanatory variables with *G*_H_2_O_ as response variables. From the full models we simplified the model by removal of each statistically non-significant explanatory variable in turn (α=0.05). We also added each removed variable back into the final reduced model one by one to assess model robustness. The initial full model and model simplification were conducted on the maximum clade credibility tree only. To assess the robustness of parameter estimates and significance to phylogenetic uncertainty, we subsequently ran the simplified model across all 1000 phylogenetic hypotheses.

For a single variable [parental feeding mode (wet/dry)], we tested the *a posteriori* hypothesis that the rate of between species diversification in *G*_H_2_O_ would be higher among species with wet incubating parents than species with dry incubating species because of constraints on *G*_H_2_O_ imposed by dry incubation. To test this hypothesis, we used the relative phenotypic rates test proposed by Thomas et al. (Thomas et al., 2006) implemented in the R library ‘motmot’. This test compares a model in which the rate of phenotypic diversification is constant lineages with a model in which rates differ between lineages with wet incubating parents and lineages with dry incubating parents. Ancestral states for incubation type were reconstructed using the ‘ace’ function in the R library ‘ape’ (Paradis et al., 2004). Note that results for the rates test were qualitatively unaffected by choice of ancestral state reconstruction methods.

### Statistical analysis

Pearson correlation coefficients (Pearson's *r*) were calculated for the mass loss between weighing sessions, across all fragments. Nested ANOVA (SAS v9.2 Proc NESTED) was conducted to partition the percentage of variability in *G*_H_2_O_ that was directly attributable to egg section (within an egg), individual egg (within a species) and individual species (within a family). We analysed whether the differences in *G*_H_2_O_ were associated with the species identity, eggshell thickness and shell section using generalised linear mixed models (SAS v9.2 Proc GLIMMIX; accounting for repeated measures from replicate fragments within an egg as a random effect). Preliminary tests confirmed that eggshell thickness and adult body mass were highly correlated with each other, with >78% of the variation explained. Therefore, in subsequent analyses, only eggshell thickness, and not adult body mass, was included. No other measures were correlated at levels >50%.

## Supplementary Material

Supplementary Material

## References

[R1] Ackerman R. A. Platter-Reiger M. (1979). Water loss by pied-billed grebe (*Podilymbus podiceps*). *Am. Zool.* 19, 921

[R2] Andersen O. Steen J. B. (1986). Water economy in bird nests. *J. Comp. Physiol. B* 156, 823-828

[R3] Ar A. Rahn H. (1978). Interdependence of gas exchange conductance, incubation length and weight of the avian egg. In *Respiratory Function in Birds, Adult and Embryonic* (ed. Piper J.), pp. 227-236 Berlin: Springer–Verlag

[R4] Ar A. Rahn H. (1980). Water in the avian egg: overall budget of incubation. *Am. Zool.* 20, 373-384

[R5] Ar A. Paganelli C. V. Reeves R. B. Greene D. G. Rahn H. (1974). The avian egg: water vapour conductance, shell thickness and functional pore area. *Condor* 76, 153-158

[R6] Arad Z. Gavrieli-Levin I. Marder J. (1988). Adaptation of the pigeon egg to incubation in dry hot environments. *Physiol. Zool.* 61, 293-300

[R7] Barrott H. G. (1937). Effect of temperature, humidity, and other factors on hatch of hens' eggs and on energy metabolism of chick embryos. Technical Bulletin, Vol. 553 Washington, DC: USDA

[R8] Benson D. A. Karsch-Mizrachi I. Lipman D. J. Ostell J. Wheeler D. L. (2007). GenBank. *Nucleic Acids Res.* 35, D21-D25 17202161 10.1093/nar/gkl986 PMC1781245

[R9] Board R. G. (1982). Properties of avian eggshells and their adaptive value. *Biol. Rev. Camb. Philos. Soc.* 57, 1-28

[R10] Booth D. T. Seymour R. S. (1987). Effect of eggshell thinning on water vapour conductance of malleefowl eggs. *Condor* 89, 453-459

[R11] Brulez K. Choudhary P. K. Maurer G. Portugal S. J. Boulton R. L. Webber S. L. Cassey P. (2014). A note on the repeatability of visually scoring eggshell patterns. *J. Ornithol.* 155, 701-706

[R12] Buhr R. J. (1995). Incubation relative humidity effects on allantoic fluid volume and hatchability. *Poult. Sci.* 74, 874-884 7603964 10.3382/ps.0740874

[R13] Carey C. (1980). Physiology of the avian egg. *Am. Zool.* 20, 325-327

[R14] Carey C. (1994). Structural and physiological differences between montane and lowland avian eggs and embryos. *J. Biosci.* 19, 429-440

[R15] Carey C. Leon-Velarde F. Dunin-Borkowski O. Bucher T. L. de la Torre G. Espinoza D. Monge C. (1989). Variation in eggshell characteristics and gas exchange of montane and lowland coot eggs. *J. Comp. Physiol. B* 159, 389-400

[R16] Carey C. Leon-Velarde F. Monge C. (1990). Eggshell conductance and other physical characteristics of avian eggs laid in the Peruvian Andes. *Condor* 92, 790-793

[R17] Cassey P. Portugal S. J. Maurer G. Ewen J. G. Boulton R. L. Hauber M. E. Blackburn T. M. (2010). Variability in avian eggshell colour: a comparative study of museum eggshells. *PLoS ONE* 5, e12054 20711258 10.1371/journal.pone.0012054 PMC2918502

[R18] Cassey P. Thomas G. Portugal S. J. Maurer G. Hauber M. Grim T. Lovell G. Miksik I. (2012). Why are birds' eggs colourful? Eggshell pigments co-vary with life-history and nesting ecology among British breeding non-passerine birds. *Biol. J. Linn. Soc. London* 106, 657-672

[R19] Cramp S. Simmons K. E. L. Perrins C. (1977-1994). *Handbook of the Birds of Europe the Middle East and North Africa – the Birds of the Western Palearctic*. Oxford: Oxford University Press

[R20] Davis T. A. Ackerman R. A. (1985). Adaptations of black tern (*Chlidonias niger*) eggs for water loss in a moist nest. *Auk* 102, 640-643

[R21] Davis T. A. Platter-Reiger M. F. Ackerman R. A. (1984). Incubation water loss by pied-billed grebe eggs: adaptation to a hot, wet nest. *Physiol. Zool.* 57, 384-391

[R22] Deeming D. C. (2002). *Avian Incubation: Behaviour, Environment and Evolution*. Oxford: Oxford University Press

[R23] Del Hoyo J. Elliot A. Sargatal J. (1992-2010). *Handbook of the Birds of the World*. Barcelona, Spain: Lynx Edicions

[R24] Drummond A. J. Rambaut A. (2007). BEAST: Bayesian evolutionary analysis by sampling trees. *BMC Evol. Biol.* 7, 214 17996036 10.1186/1471-2148-7-214 PMC2247476

[R25] Drummond A. J. Ashton B. Cheung M. Heled J. Kearse M. Moir R. Stones-Havas S. Thierer T. Wilson A. (2009). Geneious v4.8. Available at: http://www.geneious.com/

[R26] Dunning J. B. Jr (2007). *CRC Handbook of Avian Body Mass*, 2nd edn. Boca Raton, FL: CRC Press

[R27] DuRant S. E. Hepp G. R. Moore I. T. Hopkins B. C. Hopkins W. A. (2010). Slight differences in incubation temperature affect early growth and stress endocrinology of wood duck (*Aix sponsa*) ducklings. *J. Exp. Biol.* 213, 45-51 20008361 10.1242/jeb.034488

[R28] Fernandez M. S. Araya M. Arias J. L. (1997). Eggshells are shaped by a precise spatio-temporal arrangement of sequentially deposited macromolecules. *Matrix Biol.* 16, 13-20 9181550 10.1016/s0945-053x(97)90112-8

[R29] Haftorn S. (1994). The act of tremble-thrusting in tit nests, performance and possible functions. *Fauna Norvegica Series C Cinclus* 17, 55-74

[R30] Handrich Y. (1989). Incubation water loss in King penguin eggs. II. Does the brood patch interfere with eggshell conductance? *Physiol. Zool.* 62, 119-132

[R31] Hauber M. E. (2014). *The Book of Eggs*. (ed. Bates J. Becker B.). Chicago, IL: University of Chicago Press

[R32] Hoyt D. F. Board R. G. Rahn H. Paganelli C. V. (1979). The eggs of the Anatidae: conductance, pore structure and metabolism. *Physiol. Zool.* 52, 438-450

[R33] Lomholt J. P. (1976). Relationship of weight loss to ambient humidity of birds eggs during incubation. *J. Comp. Physiol.* 105, 189-196

[R34] Lomholt J. P. (1984). A preliminary study of local oxygen tensions inside bird eggs and gas exchange during early stages of embryonic development. In *Respiration and Metabolism of Embryonic Vertebrates* (ed. Seymour R. S. Junk W. Dr), pp. 289-298 Dordrecht

[R35] Maurer G. Russell D. G. D. Cassey P. (2010). Interpreting the lists and equations of egg dimensions in Schönwetter's ‘Handbuch der Oologie’. *Auk* 127, 940-947

[R36] Maurer G. Portugal S. J. Cassey P. (2011). Speckles of cryptic black-headed gull eggs show no mechanical or conductance structural function. *J. Zool.* 285, 194-204

[R37] Maurer G. Portugal S. J. Cassey P. (2012). A comparison of indices and measured values of eggshell thickness of different shell regions using museum eggs of 230 European bird species. *Ibis* 154, 714-724

[R38] Maurer G. Portugal S. J. Hauber M. E. Mikšík I. Russell D. G. D. Cassey P. (2014). First light for avian embryos: eggshell thickness and pigmentation mediate variation in development and UV exposure in wild bird eggs. *Funct. Ecol.* [Epub ahead of print] doi: 10.1111/1365-2435.12314

[R39] McNaught M. Owens I. P. F. (2002). Interspecific variation in plumage colour among birds: species isolation or light environment? *J. Evol. Biol.* 15, 505-514

[R40] Morgan S. M. Ashley-Ross M. A. Anderson D. J. (2003). Foot-mediated incubation: Nazca booby (*Sula granti*) feet as surrogate brood patches. *Physiol. Biochem. Zool.* 76, 360-366 12905122 10.1086/375430

[R41] Orme C. D. L. Davies R. G. Burgess M. Eigenbrod F. Pickup N. Olson V. Webster A. J. Ding T. S. Rasmussen P. C. Ridgely R. S. (2005). Global hotspots of species richness are not congruent with endemism or threat. *Nature* 436, 1016-1019 16107848 10.1038/nature03850

[R42] Orme C. D. L. Davies R. G. Olson V. A. Thomas G. H. Ding T. S. Rasmussen P. C. Ridgely R. S. Stattersfield A. J. Bennett P. M. Owens I. P. (2006). Global patterns of geographic range size in birds. *PLoS Biol.* 4, e208 16774453 10.1371/journal.pbio.0040208 PMC1479698

[R43] Paganelli C. V. (1980). The physics of gas exchange across the avian eggshell. *Am. Zool.* 20, 329-338

[R44] Pagel M. (1997). Inferring evolutionary processes from phylogenies. *Zool. Scr.* 26, 331-348

[R45] Pagel M. (1999). The maximum likelihood approach to reconstructing ancestral character states of discrete characters on phylogenies. *Syst. Biol.* 48, 612-622

[R46] Paradis E. Claude J. Strimmer K. (2004). APE: analyses of phylogenetics and evolution in R language. *Bioinformatics* 20, 289-290 14734327 10.1093/bioinformatics/btg412

[R47] Pérez J. H. Ardia D. R. Chad E. K. Clotfelter E. D. (2008). Experimental heating reveals nest temperature affects nestling condition in tree swallows (*Tachycineta bicolor*). *Biol. Lett.* 4, 468-471 18628112 10.1098/rsbl.2008.0266 PMC2610083

[R48] Portugal S. J. Maurer G. Cassey P. (2010a). Eggshell permeability: a standard technique for determining interspecific rates of water vapor conductance. *Physiol. Biochem. Zool.* 83, 1023-1031 20939733 10.1086/656287

[R49] Portugal S. J. Cooper H. J. Zampronio C. G. Wallace L. L. Cassey P. (2010b). Can museum egg specimens be used for proteomic analyses? *Proteome Sci.* 8, 40 20630081 10.1186/1477-5956-8-40 PMC2927511

[R50] Portugal S. J. Hauber M. E. Maurer G. Stokke B. G. Grim T. Cassey P. (2014). Rapid development of brood-parasitic cuckoo embryos cannot be explained by increased gas exchange through the eggshell. *J. Zool. (Lond.)* 293, 219-226

[R51] Rahn H. Hammel H. T. (1982). Incubation water loss, shell conductance, and pore dimensions in Adelie penguin eggs. *Polar Biol.* 1, 91-97

[R52] Rahn H. Paganelli C. V. (1990). Gas fluxes in avian eggs: driving forces and the pathway for exchange. *Comp. Biochem. Physiol.* 95A, 1-15

[R53] Rahn H. Carey C. Balmas K. Bhatia B. Paganelli C. (1977). Reduction of pore area of the avian eggshell as an adaptation to altitude. *Proc. Natl. Acad. Sci. USA* 74, 3095-3098 16592423 10.1073/pnas.74.7.3095 PMC431420

[R54] Rambaut A. (2002). Se-AI: Sequence alignment editor program, Version 2.0a11. Available at http://tree.bio.ed.ac.uk/software/seal/

[R55] Ricklefs R. E. (1969). An analysis of nesting mortality in birds. *Smithson. Contrib. Zool.* 9, 1-48

[R56] Romijn C. Roos J. (1938). The air space of the hen's egg and its changes during the period of incubation. *J. Physiol.* 94, 365-379 16995051 10.1113/jphysiol.1938.sp003687 PMC1393773

[R57] Russell D. G. D. White J. Maurer G. Cassey P. (2010). Data-poor egg collections: tapping an important research resource. *J. Afrotropical Zool.* 6, 77-82

[R58] Shackleton M. Shipman R. Ebner M. (2000). An investigation of redundant genotype-phenotype mappings and their role in evolutionary search. In *Proceedings of the 2000 Congress on Evolutionary Computation*, Vol. 1, pp. 493-500 Piscataway, NJ: IEEE Press

[R59] Sibley C. G. Monroe B. L. (1990). *Distribution and Taxonomy of Birds of the World*. New Haven, CT: Yale University Press

[R60] Skutch A. F. (1976). *Parent Birds and Their Young*. Austin, TX: University of Texas

[R61] Sotherland P. R. Packard G. C. Taigen T. L. Boardman T. J. (1980). An altitudinal cline in conductance of cliff swallow (*Petrochelidon pyrrhonota*) eggs to water vapour. *Auk* 97, 177-185

[R62] Sotherland D. P. Ashen R. M. Shuman D. Tracy C. R. (1984). The water balance of bird eggs incubated in water. *Physiol. Zool.* 57, 338-348

[R63] Thomas G. H. (2008). Phylogenetic distributions of British birds of conservation concern. *Proc. Biol. Sci.* 275, 2077-2083 18544508 10.1098/rspb.2008.0549 PMC2603218

[R64] Thomas G. H. Freckleton R. P. Székely T. (2006). Comparative analyses of the influence of developmental mode on phenotypic diversification rates in shorebirds. *Proc. Biol. Sci.* 273, 1619-1624 16769632 10.1098/rspb.2006.3488 PMC1634920

[R65] Visschedijk A. H. J. (1980). Effects of barometric pressure and abnormal gas mixtures on gaseous exchange by the avian embryo. *Am. Zool.* 20, 469-476

[R66] Vleck C. M. (1981). Hummingbird incubation: female attentiveness and egg temperature. *Oecologia* 51, 199-205 10.1007/BF00540601 28310082

[R67] Vleck C. M. Vleck D. Rahn H. Paganelli C. V. (1983). Nest microclimate, water-vapour conductance, and water loss in heron and tern eggs. *Auk* 100, 76-83

[R68] Walsberg G. E. Schmidt C. A. (1992). Effects of variable humidity on embryonic development and hatching success of mourning doves. *Auk* 109, 309-314

[R69] Yildirim I. Yetisir R. (2004). Effects of different hatcher temperatures on hatching traits of broiler embryos during the last five days of incubation. *S. Afr. J. Anim. Sci.* 34, 211-216

