# Supplementary material for: Nesting behaviour influences species-specific gas exchange across avian eggshells
Source: J Exp Biol. 2014 Sep 15;217(18):3326–32. doi: 10.1242/jeb.103291 (PMC4179895; doi:10.1242/jeb.103291)
Supplement: Supplementary Material [file supp_217_18_3326__index.html]

Supplementary Material 

# Nesting behaviour influences species-specific gas exchange across avian eggshells

## JEB103291 Supplementary Material

**Files in this Data Supplement:**

- **Supplementary Material**
