# Supplementary material for: Nesting behaviour influences species-specific gas exchange across avian eggshells
Source: J Exp Biol. 2014 Sep 15;217(18):3326–32. doi: 10.1242/jeb.103291 (PMC4179895; doi:10.1242/jeb.103291)
Supplement: Supplementary Material [file supp_217.18.3326_JEB103291.pdf]

Table S1. Full table of conductance measurements and life history traits for all species measured

Download Table S1

Table S2. Breakdown of conductance values for each egg region: B (blunt end), E (equator) and P (pointed end)

Download Table S2

Table S3. The relative contribution of the non-significant variables included in the initial full model, and which were subsequently removed

A: Species mean (water vapour conductance, G_{H2O} mg day^{-1} Torr^{-1})

Source	Df	SSQ	MS	F	*P*
Thickness	1	0.00049	0.00049	4.73	0.03
Nest	5	0.00268	0.00067	6.47	>0.001
Wet Parent	1	0.00046	0.00046	4.47	0.04
Cavity	1	0.00001	0.00001	0.13	0.72
Development	1	0.00000	0.00000	0.01	0.91
Calcium Diet	2	0.00045	0.00023	2.18	0.12
Migration	2	0.00040	0.00020	1.92	0.15
Incubation	1	0.00002	0.00002	0.22	0.64
Clutch Size	1	0.00003	0.00003	0.29	0.59
Egg Patterning	1	0.00004	0.00004	0.37	0.54
Error	122	0.01264	0.00010		

B: Blunt (water vapour conductance, G_{H2O} mg day^{-1} Torr^{-1})

Source	Df	SSQ	MS	F	*P*
Thickness	1	0.00021	0.00021	1.72	0.19
Nest	5	0.00243	0.00061	4.91	>0.001
Wet Parent	1	0.00175	0.0175	14.21	>0.001
Cavity	1	0.00001	0.00001	0.05	0.82
Development	1	0.00009	0.00009	0.73	0.39
Calcium Diet	2	0.00095	0.00048	3.85	0.02
Migration	2	0.00042	0.00021	1.72	0.18
Incubation	1	0.00008	0.00008	0.62	0.43
Clutch Size	1	0.00003	0.00003	0.24	0.62
Egg Patterning	1	0.00009	0.00009	0.75	0.39
Error	122	0.00012			
